# Cardiomyocyte Deletion of *Bmal1* Exacerbates QT- and RR-Interval Prolongation in *Scn5a*^+/Δ*KPQ*^ Mice

**DOI:** 10.3389/fphys.2021.681011

**Published:** 2021-06-24

**Authors:** Elizabeth A. Schroder, Jennifer L. Wayland, Kaitlyn M. Samuels, Syed F. Shah, Don E. Burgess, Tanya Seward, Claude S. Elayi, Karyn A. Esser, Brian P. Delisle

**Affiliations:** ^1^Department of Physiology, University of Kentucky, Lexington, KY, United States; ^2^Internal Medicine and Pulmonary, University of Kentucky, Lexington, KY, United States; ^3^CHI Saint Joseph Hospital, Lexington, KY, United States; ^4^Department of Physiology and Functional Genomics, University of Florida, Gainesville, FL, United States

**Keywords:** heart, electrophysiology, ion channel, SCN5A, long QT syndrome, *Bmal1*, *circadian*

## Abstract

Circadian rhythms are generated by cell autonomous circadian clocks that perform a ubiquitous cellular time-keeping function and cell type-specific functions important for normal physiology. Studies show inducing the deletion of the core circadian clock transcription factor *Bmal1* in adult mouse cardiomyocytes disrupts cardiac circadian clock function, cardiac ion channel expression, slows heart rate, and prolongs the QT-interval at slow heart rates. This study determined how inducing the deletion of *Bmal1* in adult cardiomyocytes impacted the *in vivo* electrophysiological phenotype of a knock-in mouse model for the arrhythmogenic long QT syndrome (*Scn5a*^+/Δ*KPQ*^). Electrocardiographic telemetry showed inducing the deletion of *Bmal1* in the cardiomyocytes of mice with or without the ΔKPQ-*Scn5a* mutation increased the QT-interval at RR-intervals that were ≥130 ms. Inducing the deletion of *Bmal1* in the cardiomyocytes of mice with or without the ΔKPQ-*Scn5a* mutation also increased the day/night rhythm-adjusted mean in the RR-interval, but it did not change the period, phase or amplitude. Compared to mice without the ΔKPQ-*Scn5a* mutation, mice with the ΔKPQ-*Scn5a* mutation had reduced heart rate variability (HRV) during the peak of the day/night rhythm in the RR-interval. Inducing the deletion of *Bmal1* in cardiomyocytes did not affect HRV in mice without the ΔKPQ-*Scn5a* mutation, but it did increase HRV in mice with the ΔKPQ-*Scn5a* mutation. The data demonstrate that deleting *Bmal1* in cardiomyocytes exacerbates QT- and RR-interval prolongation in mice with the ΔKPQ-*Scn5a* mutation.

## Introduction

Circadian rhythms alter physiology in anticipation of predictable changes in the daily environment. They are generated by cell autonomous circadian clocks in most of the cells in the body (Reppert and Weaver, 2002; Hastings et al., 2018; Michel and Meijer, 2020). Circadian clocks are formed by transcription-translation feedback loops that drive rhythmic changes in clock gene and protein expression with a periodicity of ∼24 h (Hastings et al., 2018; Michel and Meijer, 2020). The positive limb of the feedback loop is initiated by the transcription factors BMAL1 and CLOCK, which heterodimerize to activate the transcription of Period (PER) and Cryptochrome (CRY). PER and CRY proteins negatively feedback on BMAL1 and CLOCK activity. In addition to functioning as ubiquitous cellular timekeepers, circadian clocks also contribute to cell- and tissue-specific changes in physiology by regulating the expression of genes outside the timekeeping network. Studies using transgenic mouse models that allow for the selective deletion of *Bmal1* in adult cardiomyocytes show the cardiomyocyte circadian clock mechanism contributes to heart rate, ventricular repolarization and the functional expression of several cardiac ion channels (Schroder et al., 2013, 2015; Delisle et al., 2020). In this study, we determined how inducing the deletion of *Bmal1* in adult cardiomyocytes impacted cardiac electrophysiology in a genetic mouse model of long QT syndrome (LQTS).

People living with congenital LQTS have a high risk for ventricular arrhythmias that can lead to syncope, seizures, and/or sudden cardiac death (SCD) (Schwartz et al., 1993; Moss, 2003). Most cases of LQTS are caused by mutations in one of three different cardiac ion channel genes (LQT1-LQT3). LQT3 is caused by mutations in the predominant voltage-gated Na+ channel expressed in the heart (*SCN5A*/Nav1.5). Some LQT3-linked mutations associate with more than one type of arrhythmia syndrome/phenotype in people, including cardiac conduction defects, atrial arrhythmias, and/or Brugada Syndrome (Remme, 2013). People who have the LQT3-linked *SCN5A* deletion mutation ΔKPQ1505-1507 can suffer LQT3 and cardiac conduction defects (Zareba et al., 2001). Heterozygous knock-in mice with the equivalent ΔKPQ-*Scn5a* mutation (*Scn5a*^+/Δ*KPQ*^) have abnormally long QT- and RR-intervals (Nuyens et al., 2001; Fabritz et al., 2010; Lemoine et al., 2011; Wu et al., 2012). We tested the hypothesis that inducing the deletion of *Bmal1* in *Scn5a*^+/Δ*KPQ*^ mice would exacerbate QT- and RR-interval prolongation using electrocardiographic (ECG) telemetry.

## Materials and Methods

### Animals

All animal procedures were conducted in compliance with the guidelines of the Association for Assessment and Accreditation of Laboratory Animal Care and were approved by the Institutional Animal Care and Use Committee at University of Kentucky. Mice for these studies were bred by crossing the floxed *Bmal1* (*Bmal1*^*f/f*^) mouse and the cardiac-specific, *Myh6-MerCreMer* recombinase mouse (iCSΔ*Bmal1*) (Schroder et al., 2013). The *Scn5a*^+/Δ*KPQ*^ mice (kindly provided by Dr. Peter Carmeliet) were bred with the iCSΔ*Bmal1* mice to generate an B6Cre^±^;B6*Bmal1*^*f/f*^;129*Scn5a*^+/Δ*KPQ*^ (iCSΔ*Bmal1/Scn5a*^+/Δ*KPQ*^) mouse. The iCSΔ*Bmal1*^+/+^ mice consisted of vehicle injected mice or iCSΔ*Bmal1* mice assessed prior to tamoxifen injection. *Cre*-recombination was activated by intraperitoneal injections of tamoxifen (2 mg/day) for 5 consecutive days to generate iCSΔ*Bmal1*^–/–^ mice. The concentration and duration of the tamoxifen injections used have been shown to cause effective cardiomyocyte-specific recombination without any obvious long-term tamoxifen toxicity as assessed by changes in the structure, function, and the ECG (Eckardt et al., 2004; Andersson et al., 2009; Hall et al., 2011; Schroder et al., 2013). Mice were housed in 12 h light and 12 h dark cycles with *ad libitum* access to food and water.

### Circadian Collections

mRNA collections were done as described previously (Schroder et al., 2013). Male and female iCSΔ*Bmal1* mice at 14–16 weeks of age were housed in light boxes and entrained to a 12:12-h light-dark cycle. Two weeks after the final injection of vehicle (32 mice) or tamoxifen (32 mice), mice were released into constant darkness. After 30 h in darkness (18 h after the beginning of the subjective light phase, Circadian Time or CT = 18 h), mice were euthanized under dim red light (< 5 lux) and hearts were collected every 4 h for 28 h, a total of 8 time points. RNA was prepared for quantitative PCR (qtPCR) using TaqMan (Applied Biosystems) assays to examine transcript expression. The ΔΔCT method was used for the quantification of qtPCR data. Gene expression is shown as the relative value compared with the mean vehicle value.

### ECG Telemetry

*In vivo* ECG telemetry was performed as described previously (Schroder et al., 2013, 2014). Briefly, male mice at 14 weeks of age were anesthetized with isoflurane and transmitter units (DSI, TA11ETA-F10) were implanted in the peritoneal cavity. The two ECG leads were secured near the apex of the heart and the right acromion. Mice were housed singly and allowed to recover for 1 week before beginning recording. Telemetry data were recorded at 1000 Hz before and after the intraperitoneal injection of tamoxifen to activate deletion of *Bmal1* by *Cre-*recombination. After injection, mice were given at least 4 weeks to recover before ECG data were recorded.

Ponemah telemetry software (Data Science International) was used to quantify QT-intervals. QT-intervals were analyzed at RR-intervals ranging from 90 ± 3 ms to 140 ± 3 ms in 10 ms bins. Two independent investigators performed these analyses.

Ponemah was also used to measure RR-intervals and HRV. We plotted 3-days of averaged RR-intervals (15-min averages) and fit the individual data with the following cosine function:

I⁢n⁢t⁢e⁢r⁢v⁢a⁢l=A*c⁢o⁢s⁢(2⁢π⁢(t-τ)/T)+m

This allowed calculation of the period (T), phase (τ), amplitude of the oscillation (A), and rhythm-adjusted mean (m).

HRV was analyzed using the time domain parameters for the standard deviation of all RR-intervals in sinus rhythm (SDNN, in ms); the root mean square differences between successive RR-intervals (RMSSD, in ms); and the percentage of normal consecutive RR-intervals differing by ≥ 6 ms similar to that previously described (Thireau et al., 2008; Fenske et al., 2016; Moghtadaei et al., 2017). SDNN is an index of total sinus rhythm HRV, whereas RMSSD is a beat to beat index of HRV and reflects abrupt changes in RR-intervals. PNN6 is thought to reflect HRV secondary to parasympathetic tone. Each parameter was measured in 15-min episodes. Individual mouse data sets were not well described using a cosine function, so we compared averaged values that corresponded to 2–5, 8–11, 14–17, or 20–23 h after the start of the light phase (Zeitgeber time or ZT = 2–5, 8–11, 14–17, or 20–23 h, respectively).

### Statistical Analysis

The data were analyzed using a two-way ANOVA to identify significant interactions (PRISM, MathWorks). We used the Šidák correction method to correct for multiple comparisons. For gene expression studies, the statistical JTK_CYCLE package was used to identify mRNA transcripts that had circadian expression profiles (Hughes et al., 2010).

## Results

### Inducing the Deletion of *Bmal1* in Adult Cardiomyocyte Decreases the mRNA Transcript Levels for Several Cardiac Ion Channels and Transcription Factors Important for Repolarization and Conduction

Studies suggest that *Bmal1* directly and indirectly contributes to the transcription of cardiac ion channel genes *Scn5a*, *Kcnh2*, *Hcn4*, and *Kchip2* (Jeyaraj et al., 2012; Schroder et al., 2013, 2015; D’Souza et al., 2020). *Bmal1* may regulate cardiac ion channel transcription by binding to enhancer box (E-box) elements in the promoters of ion channel genes, or *Bmal1* can modify the expression of other transcription factors that regulate ion channel transcription. We wanted to determine how inducing the deletion of *Bmal1* in the adult heart impacts the expression profiles for a large number of candidate ion channel genes that encode proteins important for cardiac depolarization, repolarization, conduction, and ion channel transcription in humans and mice (London, 2001; Eckardt et al., 2004; Nerbonne and Kass, 2005; Arnolds et al., 2012; Herrmann et al., 2012; Jeyaraj et al., 2012; Mao et al., 2012; Cai et al., 2014; Nerbonne, 2014; Wahl-Schott et al., 2014; Tarradas et al., 2017).

In mice and humans, the cardiac Na^+^ current (I_*Na*_) generates the rapid upstroke of the action potential in the working myocardium and the funny current (I_*F*_) initiates depolarization in the autorhythmic myocardium. *Scn5a and Hcn4* encode the major pore-forming proteins that conduct I_*Na*_ and I_*F*_ (Abriel, 2007; Kozasa et al., 2018; D’Souza et al., 2020). Also similar to humans, the inward rectifier K^+^ current (I_*K*1_) in the mouse working myocardium is responsible for stabilizing the resting membrane potential. *Kcnj2* encodes a critical pore-forming subunit that conducts I_*K*1_ (Nerbonne and Kass, 2005; Nerbonne, 2014). Unlike humans, the rapid and slowly activating delayed rectifier K^+^ currents (I_*Ks*_ and I_*Kr*_, respectively) are not major contributors to ventricular repolarization in mice. However, the genes that encode the pore-forming proteins for I_*Ks*_ and I_*Kr*_, *Kcnq1*, and *Kcnh2*, are expressed in the mouse myocardium (Pond et al., 2000; Knollmann et al., 2007). Ventricular repolarization in mice is regulated by several K^+^ currents, including the transient outward K^+^ currents (I_*to*_) and the slow K^+^ currents (I_*K,slow*_). *Kcnd2*, *Kchip2*, *Kcna5*, and *Kcnb1* encode important pore-forming and auxiliary ion channel proteins that contribute to I_*to*_ and I_*K,slow*_ (Nerbonne and Kass, 2005; Nerbonne, 2014). *Gja1*, *Gja5*, and *Gjc1* encode the cardiac gap junction proteins (connexins) that mediate conduction between cardiomyocytes (Jalife et al., 1999). The *Scn4b* auxiliary Na^+^ channel subunit is a genetic modifier of cardiac conduction speed in mouse models of Na^+^ channelopathies (Remme et al., 2009). The transcription factor genes *Klf15*, *Tbx5*, *Gata4*, and *Foxo1* regulate the expression of certain cardiac ion channel genes, including *Kchip2* or *Scn5a*, previous studies show that these transcription factors have circadian expression profiles in the mouse heart (Arnolds et al., 2012; Jeyaraj et al., 2012; Mao et al., 2012; Pizarro et al., 2013; Cai et al., 2014; Tarradas et al., 2017).

We previously reported the *Scn5a, Kcnh2*, and *Kcnd2* mRNA transcript expression profiles in iCSΔ*Bmal1*^+/+^ and iCSΔ*Bmal1*^–/–^ mouse hearts (Schroder et al., 2013, 2015). We found that *Scn5a*, *Kcnh2 and Kcnd2* had circadian expression profiles in iCSΔ*Bmal1*^+/+^ mouse hearts. The circadian expression profiles for *Scn5a* and *Kcnh2* (but not *Kcnd2*) were lost after inducing the deletion of *Bmal1* (iCSΔ*Bmal1*^–/–^), and, compared to mRNA transcript levels in iCSΔ*Bmal1*^+/+^ mouse hearts, the mRNA transcript levels for *Scn5a* and *Kcnh2* (but not *Kcnd2*) were reduced in iCSΔ*Bmal1*^–/–^ mice.

**FIGURE 1:**
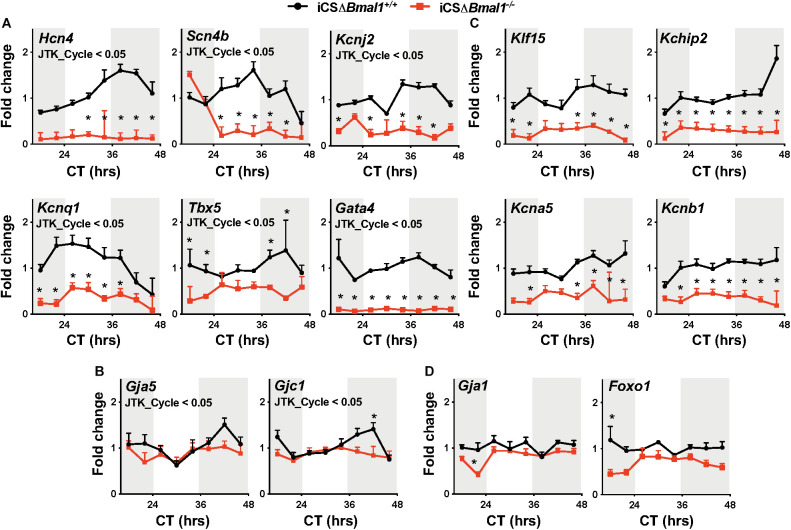
Inducing the deletion of *Bmal1* in adult cardiomyocytes differentially impacts the temporal expression of cardiac ion channel transcripts. Shown are qPCR profiles for mRNA transcripts measured from iCSΔ*Bmal1*^+/+^ (black circles) and iCSΔ*Bmal1*^–/–^ (red squares) mouse hearts plotted as a function of circadian time (CT). Light and shaded regions on x-axis represent subjective light and dark cycles. **(A)** *Hcn4*, *Scn4b, Kcnj2*, *Kcnq1*, *Tbx5*, and *Gata4* mRNA transcripts had circadian expression profiles in the hearts of iCSΔ*Bmal1*^+/+^ mice (JTK_Cycle < 0.05) but not iCSΔ*Bmal1*^–/–^ mice. mRNA expression was lower at most time points in the hearts of iCSΔ*Bmal1*^–/–^ mice when compared to iCSΔ*Bmal1*^+/+^ mouse hearts (*n* = 3–4/time point; *p < 0.05). **(B)** *Gja5* and *Gjc1* mRNA transcripts had circadian expression profiles in iCSΔ*Bmal1*^+/+^ mouse hearts (JTK_Cycle < 0.05) but not iCSΔ*Bmal1*^–/–^mouse hearts. The overall transcript levels were similar in iCSΔ*Bmal1*^+/+^ and iCSΔ*Bmal1*^–/–^ mice at most circadian time points (*n* = 3-4/time point; *p < 0.05). **(C)** *Klf15*, *Kchip2*, *Kcna5*, and *Kcnb1* mRNA transcripts did not have circadian expression profiles in the hearts of iCSΔ*Bmal1*^+/+^ or iCSΔ*Bmal1*^–/–^ mice. (JTK_Cycle > 0.05). The overall transcript levels were lower in iCSΔ*Bmal1*^–/–^ mouse hearts compared to transcript levels in iCSΔ*Bmal1*^+/+^ mouse hearts at most circadian time points (*n* = 3–4/time point; *p < 0.05). **(D)** *Gja1* and *Foxo1* mRNA transcripts did not have circadian expression profiles in the hearts of iCSΔ*Bmal1*^+/+^ or iCSΔ*Bmal1*^–/–^ mice (JTK_Cycle > 0.05) and the overall transcript levels were similar at most circadian time points (*n* = 3–4/time point; *p < 0.05).

In this study, *Hcn4*, *Kcnj2*, *Kcnq1*, *Kchip2*, *Kcna5*, *Kcnb1*, *Gja1*, *Gja5*, *Gjc1*, *Scn4b*, *Klf15*, *Tbx5*, *Gata4*, and *Foxo1* transcripts were assessed for both the circadian expression and overall expression level by quantitative PCR. These data demonstrate that *Hcn4, Kcnj2, Kcnq1, Scn4b*, *Tbx5*, *Gata4, Gja5*, and *Gjc1* mRNA transcript levels have circadian expression profiles in the hearts of iCSΔ*Bmal1*^+/+^ mice but not in iCSΔ*Bmal1*^–/–^ mice (Figures 1A,B). Compared to mRNA transcript levels in iCSΔ*Bmal1*^+/+^ mouse hearts, the mRNA transcript levels in the hearts of iCSΔ*Bmal1*^–/–^ mice for *Hcn4, Kcnj2, Kcnq1, Scn4b*, *Gata4*, and *Tbx5* (but not *Gja5* or *Gjc1*) were reduced.

*Klf15*, *Kchip2*, *Kcna5*, *Kcnb1*, *Gja1*, and *Foxo1* mRNA transcripts did not show a circadian expression profile in the hearts of iCSΔ*Bmal1*^+/+^ or iCSΔ*Bmal1*^–/–^ mice (Figures 1C,D). However, compared to mRNA transcript levels in the hearts of iCSΔ*Bmal1*^+/+^ mice, the mRNA transcript levels for *Klf15*, *Kchip2*, *Kcna5*, and *Kcnb1* in iCSΔ*Bmal1*^–/–^ mice were reduced (Figure 1C). The mRNA transcript levels for *Gja1* and *Foxo1* in iCSΔ*Bmal1*^+/+^ and iCSΔ*Bmal1*^–/–^ mice were not different (Figure 1D). Together, these findings demonstrate that the loss of *Bmal1* in adult cardiomyocytes causes a loss of circadian expression and/or a reduction in overall mRNA transcript levels for several cardiac ion channels and transcription factors important for cardiac depolarization, repolarization and conduction.

### Inducing the Deletion of *Bmal1* Increases the QT-Interval at Slow Heart Rates

**FIGURE 2:**
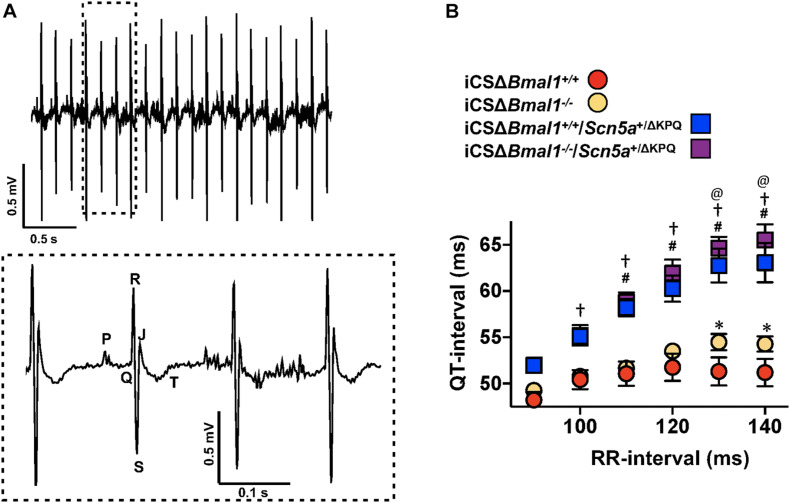
Deletion of *Bmal1* in adult cardiomyocytes prolongs the QT-interval at longer RR-intervals. **(A)** Representative electrocardiogram trace measured from the mice used in this study. The inset shows the dashed box region on the ECG trace in more detail to highlight the P, Q, R, S, J, and T waves. **(B)** The mean QT-interval plus standard error of the mean measured between 90 and 140 ms in iCSΔ*Bmal1*^+/+^ (red circles), iCSΔ*Bmal1*^+/+^*/Scn5a*^+/Δ*KPQ*^ (blue squares), iCSΔ*Bmal1*^–/–^ (orange circles) or iCSΔ*Bmal1*^–/–^*/Scn5a*^+/Δ*KPQ*^ (purple squares) mice is shown (*n* = 6 and *n* = 8, respectively). We compared QT intervals at each RR-interval between iCSΔ*Bmal1*^+/+^ and iCSΔ*Bmal1*^+/+^*/Scn5a*^+/Δ*KPQ*^ (^#^*p* < 0.05); iCSΔ*Bmal1*^–/–^ or iCSΔ*Bmal1*^–/–^*/Scn5a*^+/Δ*KPQ*^ (^†^*p* < 0.05); iCSΔ*Bmal1*^+/+^ and iCSΔ*Bmal1*^–/–^ (*p < 0.05); and iCSΔ*Bmal1*^+/+^*/Scn5a*^+/Δ*KPQ*^ and iCSΔ*Bmal1*^–/–^*/Scn5a*^+/Δ*KPQ*^ (^@^*p* < 0.05).

The changes in cardiac ion channel expression that occurred after inducing the deletion of *Bmal1* in adult cardiomyocytes motivated us to determine how it would impact the cardiac electrophysiological phenotype of mice with the ΔKPQ-*Scn5a* mutation. We used ECG telemetry to measure QT- and RR-intervals in conscious free moving mice housed in 12-h light and 12-h dark cycles prior to and after inducing *Bmal1* deletion in cardiomyocytes (Figure 2A). Previous studies show that the QT-intervals measured from wildtype C57BL/6 mice do not depend on the preceding RR-intervals (Drici et al., 1998; Speerschneider and Thomsen, 2013; Roussel et al., 2016). We confirmed these findings in the iCSΔ*Bmal1*^+/+^ mice by measuring the QT-intervals at RR-intervals ranging from 90 to 140 ms in 10 ms bins (Figure 2B). Deletion of *Bmal1* in cardiomyocytes caused the QT-intervals to become longer at slower RR-intervals. Compared to iCSΔ*Bmal1*^+/+^ mice, the iCSΔ*Bmal1*^–/–^ mice had longer QT-intervals at RR-intervals that were ≥ 130 ms.

QT-intervals in mice with the ΔKPQ-*Scn5a* mutation were dependent on the duration of preceding RR-intervals (Figure 2B). Compared to the QT-intervals measured in the iCSΔ*Bmal1*^+/+^ mice, the QT-intervals in iCSΔ*Bmal1*^+/+^*/Scn5a*^+/Δ*KPQ*^ mice were longer at RR-intervals that were ≥ 110 ms. Compared to the iCSΔ*Bmal1*^–/–^ mice, the iCSΔ*Bmal1*^–/–^*/Scn5a*^+/Δ*KPQ*^ mice had longer QT-intervals at RR-intervals that were ≥ 100 ms. Inducing the deletion of *Bmal1* in mice with the ΔKPQ-*Scn5a* mutation also increased the QT-interval at RR-intervals that were ≥ 130 ms. These data demonstrate that *Bmal1* deletion in adult cardiomyocytes prolongs the QT-interval at slow heart rates in mice with or without the ΔKPQ-*Scn5a* mutation.

### Inducing the Deletion of *Bmal1* Increases the Day/Night Rhythm-Adjusted Mean in *the* RR-Interval in Mice With or Without the ΔKPQ-*Scn5a* Mutation

**FIGURE 3:**
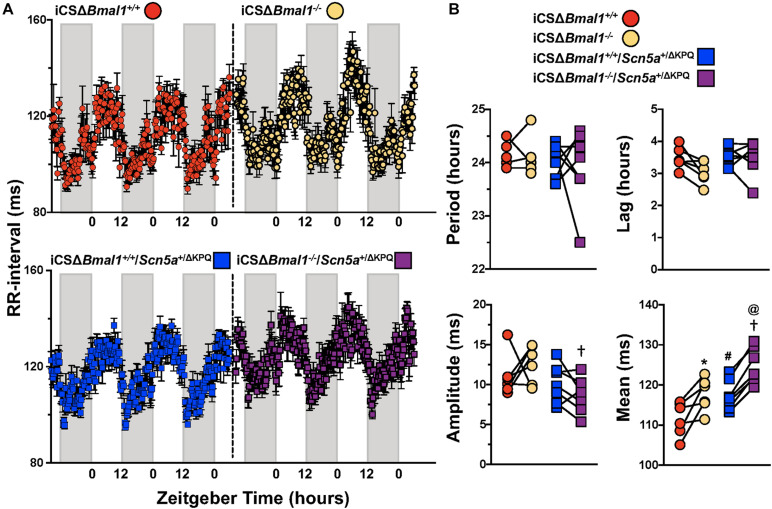
Deletion of *Bmal1* in adult cardiomyocytes increases the rhythm adjusted mean of the RR-interval. **(A)** RR intervals were averaged in 15-min bins over 3 days for iCSΔ*Bmal1*^+/+^ (red circles) and iCSΔ*Bmal1*^–/–^ (orange circles) (top graph, *n* = 6) or iCSΔ*Bmal1*^+/+^*/Scn5a*^+/Δ*KPQ*^ (blue circles) and iCSΔ*Bmal1*^–/–^*/Scn5a*^+/Δ*KPQ*^ (purple circles) mice (bottom graph, *n* = 7). Gray shaded regions on x-axis represent lights off. **(B)** The data for each mouse were fit to cosine curves to analyze period, lag, amplitude and median. The graphical representation of the change in period, amplitude and slope for iCSΔ*Bmal1*^+/+^ (red circles) and iCSΔ*Bmal1*^–/–^ (orange circles) and iCSΔ*Bmal1*^+/+^*/Scn5a*^+/Δ*KPQ*^ (blue squares) and iCSΔ*Bmal1*^–/–^*/Scn5a*^+/Δ*KPQ*^ (purple squares) mice are shown. Analysis showed that the rhythm adjusted mean between iCSΔ*Bmal1*^+/+^ and iCSΔ*Bmal1*^+/+^*/Scn5a*^+/Δ*KPQ*^ (^#^*p* < 0.05) were different; the amplitude and rhythm adjusted mean for iCSΔ*Bmal1*^–/–^ or iCSΔ*Bmal1*^–/–^*/Scn5a*^+/Δ*KPQ*^ were different (^†^*p* < 0.05); the rhythm adjusted mean for iCSΔ*Bmal1*^+/+^ and iCSΔ*Bmal1*^–/–^ were different (*p < 0.05); and the rhythm adjusted mean for iCSΔ*Bmal1*^+/+^*/Scn5a*^+/Δ*KPQ*^ and iCSΔ*Bmal1*^–/–^*/Scn5a*^+/Δ*KPQ*^ were different (^@^*p* < 0.05).

We quantified the day/night rhythm in the RR-interval recorded from mice before and after inducing the deletion of *Bmal1* in adult cardiomyocytes with or without the ΔKPQ-*Scn5a* mutation. Mice were housed in 12 h light and 12 h dark cycles. The RR-intervals were averaged every 15-min and plotted as a function of ZT for 3 days (Figure 3A). The individual data from each mouse were fit with a cosine function to calculate the day/night period, acrophase (the ZT at which the rhythms peaked), amplitude, and rhythm adjusted mean (Figure 3B). The day/night rhythm in the RR-intervals had periods that were ∼24 h in mice before and after deletion of *Bmal1* in cardiomyocytes with or without the ΔKPQ-*Scn5a* mutation. The day/night rhythm in the RR-intervals peaked after the beginning of the light cycle and did not change after deletion of *Bmal1* in the cardiomyocytes of mice with or without the ΔKPQ-*Scn5a* mutation. The amplitudes in the day/night rhythm of the RR-intervals were not different before or after deletion of *Bmal1* in cardiomyocytes in both groups of mice, however, the iCSΔ*Bmal1*^–/–^ mice had larger amplitudes compared to iCSΔ*Bmal1*^–/–^*/Scn5a*^+/Δ*KPQ*^ mice. The biggest differences among the groups of mice were in day/night rhythm-adjusted means of the RR-intervals. Compared to iCSΔ*Bmal1*^+/+^ mice, the rhythm-adjusted means for the RR-intervals were longer in iCSΔ*Bmal1*^+/+^*/Scn5a*^+/Δ*KPQ*^ mice. Inducing the deletion of *Bmal1* in cardiomyocytes increased the rhythm-adjusted means for both groups of mice by similar amounts (Figure 3B). These data demonstrate that deletion of *Bmal1* in cardiomyocytes does not impact the period, phase or amplitude in the day/night rhythm in RR-intervals in mice with or without the ΔKPQ-*Scn5a* mutation. Deletion of *Bmal1* in adult cardiomyocytes prolongs the day/night rhythm-adjusted mean in the RR-intervals of mice with or without the ΔKPQ-*Scn5a* mutation.

### Mice With the ΔKPQ-*Scn5a* Mutation Have Reduced HRV That Is Normalized After Inducing the Deletion of *Bmal1*

**FIGURE 4:**
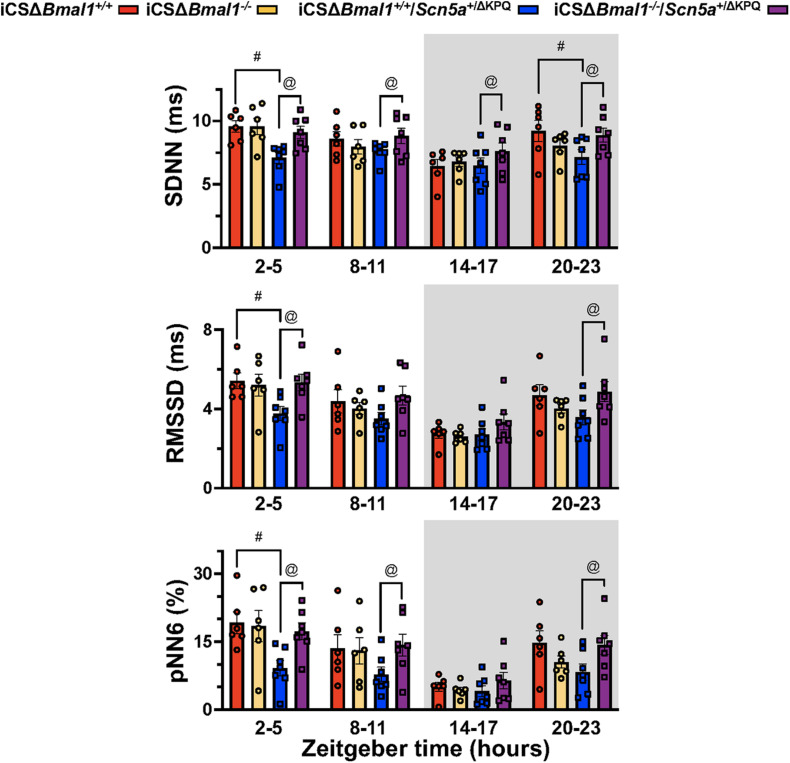
Mice with the ΔKPQ-*Scn5a* mutation have reduced HRV and this is increased by inducing the deletion of *Bmal1* in adult cardiomyocytes. The graphical representation of the change SDNN (top graph), RMSD (middle graph) or pNN6 (bottom graph) for iCSΔ*Bmal1*^+/+^ (red bars, circles), iCSΔ*Bmal1*^–/–^ (orange bars, circles), or iCSΔ*Bmal1*^+/+^*/Scn5a*^+/Δ*KPQ*^ (blue bars, circles), and iCSΔ*Bmal1*^–/–^*/Scn5a*^+/Δ*KPQ*^ (purple bars, circles) mice measured at ZT = 2–5, 8–11, 14–17, and 20–23 h are shown (*n* = 6 and *n* = 7, respectively). Analysis detected differences in the SDNN, RMSSD and PNN6 between iCSΔ*Bmal1*^+/+^ and iCSΔ*Bmal1*^+/+^*/Scn5a*^+/Δ*KPQ*^ mice (^#^*p* < 0.05) at certain timepoints, as well as differences in the SDNN, RMSSD and PNN6 between iCSΔ*Bmal1*^+/+^*/Scn5a*^+/Δ*KPQ*^ and iCSΔ*Bmal1*^–/–^*/Scn5a*^+/Δ*KPQ*^ mice (^@^*p* < 0.05).

Heart rate variability measures changes in sinoatrial node function secondary to autonomic drive, autonomic sensitivity of the sinoatrial nodal cells, and/or changes in the spontaneous depolarization of SAN cells (Fenske et al., 2016). We tested the hypothesis that the HRV in the iCSΔ*Bmal1*^+/+^*/Scn5a*^+/Δ*KPQ*^ mice was different than the iCSΔ*Bmal1*^+/+^ mice. We measured the 3-day average in the SDNN, RMSSD, and pNN6 at ZT = 2-5 h, ZT = 8–11 h, ZT = 14–17 h, or ZT = 20–23 h. We found that when RR-intervals were longest (ZT = 2–5 h), the SDNN, RMSSD and pNN6 were smaller in the iCSΔ*Bmal1*^+/+^*/Scn5a*^+/Δ*KPQ*^ mice compared to iCSΔ*Bmal1*^+/+^ mice (Figure 4). Compared to the iCSΔ*Bmal1*^+/+^ mice, the HRV did not change in the iCSΔ*Bmal1*^–/–^ mice at any of the timepoints tested. Compared to iCSΔ*Bmal1*^+/+^*/Scn5a*^+/Δ*KPQ*^ mice, the iCSΔ*Bmal1*^–/–^*/Scn5a*^+/Δ*KPQ*^ mice had SDNN values that increased at all the timepoints tested; RMSSD values that increased at ZT = 2–5 h and ZT = 20–23 h; and pNN6 values that increased at ZT = 2–5 h, ZT = 8–11 h, and ZT = 20–23 h. The data demonstrate that inducing the deletion of *Bmal1* in the cardiomyocytes of mice with the ΔKPQ-*Scn5a* mutation normalize the HRV to levels similar to those seen in iCSΔ*Bmal1*^+/+^ or iCSΔ*Bmal1*^–/–^ mice.

## Discussion

This study showed that the deletion of *Bmal1* in adult cardiomyocytes disrupts the expression profiles for a number of cardiac ion channel and transcription factor transcripts important for normal cardiac repolarization, depolarization, and conduction. We tested the hypothesis that the deletion of *Bmal1* in adult cardiomyocytes would modify the cardiac electrophysiological phenotype in mice that harbor the ΔKPQ-*Scn5a* mutation *in vivo*. We found that the mice with the ΔKPQ-*Scn5a* mutation had abnormally long QT-intervals, steeper QT- and RR-interval relations, slower day/night rhythm-adjusted means in RR-intervals and decreased HRV. Inducing the deletion of *Bmal1* in the hearts of mice with the ΔKPQ-*Scn5a* mutation prolonged the QT-intervals measured at slower RR-intervals and increased the day/night rhythm-adjusted mean in RR-interval. The absolute magnitude of these changes was similar in mice without the ΔKPQ-*Scn5a* mutation, suggesting that the changes caused by deletion of *Bmal1* in cardiomyocytes were additive. The deletion of *Bmal1* in cardiomyocytes did not alter HRV in mice without the ΔKPQ-*Scn5a* mutation, but it increased the abnormally low HRV in mice with the ΔKPQ-*Scn5a* mutation. We conclude that deletion of *Bmal1* in cardiomyocytes disrupts the expression for a number of cardiac ion channel genes, prolongs the QT- and RR-intervals in mice with or without the ΔKPQ-*Scn5a* mutation, and normalizes HRV in mice with the ΔKPQ-*Scn5a* mutation.

### *Bmal1* Is Important for Normal Ventricular Repolarization at Slow Heart Rates

Inducing the deletion of *Bmal1* prolongs the QT-intervals at slower heart rates in mice with and without the ΔKPQ-*Scn5a* mutation (Figure 2). This might not have been expected in mice with the ΔKPQ-*Scn5a* mutation since previous studies show that inducing the deletion of *Bmal1* in adult cardiomyocytes decreases the functional expression of *Scn5a* by ∼30% (Schroder et al., 2013). We now show that *Bmal1* directly or indirectly contributes to the expression of many different cardiac transcripts that regulate normal depolarization, repolarization and conduction in mice (Figure 1; Jeyaraj et al., 2012; Schroder et al., 2015; Delisle et al., 2020). Thus, the cardiac electrophysiological properties likely do not reflect the changes in one gene but rather many different genes.

### The Day/Night Rhythm in Heart Rate *in vivo* Does Not Depend on the Cardiomyocyte Circadian Clock

Inducing the deletion of *Bmal1* did not change the phase, amplitude or period in the day/night rhythm of heart rate in mice with or without ΔKPQ-*Scn5a* mutation (Figure 3; Schroder et al., 2013). These data provide additional support that the day/night rhythm in heart rate *in vivo* does not depend on the functional circadian clock mechanism in cardiomyocytes but rather other factors including the autonomic nervous system, nocturnal behavioral patterns, core body temperature, circadian rhythms in neurohumoral signaling, etc (Sheward et al., 2010; Tong et al., 2013; Schroder et al., 2014). This study confirms previous findings that the deletion of *Bmal1* in adult cardiomyocytes slows the day/night rhythm-adjusted mean in heart rate (Schroder et al., 2013). The slowing of the mean heart rate after inducing the deletion of *Bmal1* was observed in mice with or without the ΔKPQ-*Scn5a* mutation.

### The Reduction in HRV in Mice With ΔKPQ-*Scn5a* Is Not Secondary to Slower Heart Rates

Another novel finding in this study is that, compared to mice without the ΔKPQ-*Scn5a* mutation, mice with the ΔKPQ-*Scn5a* mutation had a lower HRV when heart rate was slowest. The lower HRV likely reflects sinoatrial node dysfunction, changes in the autonomic sensitivity of the sinoatrial node and/or changes in autonomic tone (Fabritz et al., 2010; Wu et al., 2012). Although studies have not shown changes in the autonomic sensitivity of the sinoatrial node in *Scn5a*^+/Δ*KPQ*^ mice, studies do show that, compared to WT mice, the working myocardium of *Scn5a*^+/Δ*KPQ*^ mice expresses fewer β-adrenergic receptors (Fabritz et al., 2010). Another possibility is that the reduction in HRV measured in the iCSΔ*Bmal1*^+/+^*/Scn5a*^+/Δ*KPQ*^ mice reflects differences in basal heart rate compared to the iCSΔ*Bmal1*^+/+^ mice. However, this seems unlikely because HRV increases as the heart rates become slower (Monfredi et al., 2014). The increase in HRV normally seen at slower heart rates might explain why HRV increased in the iCSΔ*Bmal1*^–/–^*/Scn5a*^+/Δ*KPQ*^ mice (Figures 3, 4). Another possible explanation for the effect on HRV is the recent observation that the loss of *Bmal1* in the heart alters the intrinsic beating rate of sinoatrial node preparations (D’Souza et al., 2020). The time of day changes in sinoatrial node intrinsic beating rate were suggested to be secondary to a reduction in the functional expression of the *Hcn4*. Consistent with these studies, we found that cardiac *Hcn4* mRNA transcripts in the hearts of iCSΔ*Bmal1*^+/+^ mice had a circadian expression profile that was lost and reduced iCSΔ*Bmal1*^–/–^ mice (Figure 1). Whether or not the decreased expression of *Hcn4* mRNA transcript levels and/or other cardiac ion channel genes is responsible for the slower rhythm-adjusted mean in heart rate or increased HRV in mice with the ΔKPQ-*Scn5a* mutation warrants further investigation.

### Study Limitations

There are several limitations to this study. Mouse models are widely employed by cardiac electrophysiologists because they are practical for determining how genetic, pharmacological and/or environmental manipulations impact arrhythmogenic triggers and/or pro-arrhythmic changes in the cardiac substrate (Dobrev and Wehrens, 2018; Clauss et al., 2019). However, there are clear species-specific differences in cardiac electrophysiology that limit extrapolation to humans (Sabir et al., 2008; Nerbonne, 2014). Mouse hearts are small and beat about 10 times faster than humans. Although human and mouse ECG waveforms have P, QRS, and T-waves, mouse hearts repolarize quickly to generate a predominant J wave and a small inverted T-wave (Mitchell et al., 1998; Kaese and Verheule, 2012; Boukens et al., 2014). As such, the underlying mechanisms for cardiac excitability and arrhythmias in mouse and human hearts are different (Sabir et al., 2008).

We did not observe any obvious spontaneous ventricular symptoms (e.g., ventricular tachyarrhythmias) in iCSΔ*Bmal1*^+/+^/*Scn5a*^+/Δ*KPQ*^ or iCSΔ*Bmal1*^+/+^/*Scn5a*^+/Δ*KPQ*^ mice during the ECG recordings (∼6-9 days). Therefore, we cannot conclude that inducing the deletion of *Bmal1* in cardiomyocytes increases arrhythmogenicity in mice with the ΔKPQ-*Scn5a* mutation. However, similar to the Nuyens et al., 2001 study (Nuyens et al., 2001), we did see that mice harboring the ΔKPQ-*Scn5a* mutation had a higher number of sinus pauses, atrioventricular block and bradyarrhythmias. The absolute number of hourly pauses/blocks during the inactive phase trended higher in the iCSΔ*Bmal1*^–/–^/*Scn5a*^+/Δ*KPQ*^ mice compared to the iCSΔ*Bmal1*^+/+^/*Scn5a*^+/Δ*KPQ*^ mice but was not significant (iCSΔ*Bmal1*^+/+^/*Scn5a*^+/Δ*KPQ*^ = 6.7 ± 2.5 pauses/blocks per hour vs iCSΔ*Bmal1*^–/–^/*Scn5a*^+/Δ*KPQ*^ = 20.3 ± 7.8 pauses/blocks per hour, *n* = 6 mice, p = 0.06). More invasive studies are needed to determine whether the loss of the *Bmal1* in mice with the ΔKPQ-*Scn5a* mutation have higher susceptibility to arrhythmias and/or ventricular tachyarrhythmias.

### Conclusion

In summary, this study shows inducing the deletion of *Bmal1* in mouse hearts exacerbates the prolongation in the QT- and RR-intervals in mice with the ΔKPQ-*Scn5a* mutation. Specifically, it increases the abnormal prolongation in the QT-intervals at slower heart rates and causes an overall slowing of the heart rate over 24-h. These effects combine to increase the absolute number of abnormal QT-intervals during the 24-h cycle. However, due to the low absolute probability of ventricular symptoms, we cannot conclude it increases the likelihood of ventricular tachyarrhythmias. Future studies that explore the molecular mechanisms with which *Bmal1* functions to limit QT-interval prolongation at slow heart rates might lead to the identification of novel therapeutic targets.

## Data Availability Statement

The raw data supporting the conclusions of this article will be made available by the authors, without undue reservation.

## Ethics Statement

The animal study was reviewed and approved by Association for Assessment and Accreditation of Laboratory Animal Care and was approved by the Institutional Animal Care and Use Committee at University of Kentucky.

## Author Contributions

ES, KE, CE, and BD contributed and developed the research design. ES, JW, KS, SS, DB, and BD worked on data acquisition and analyses. ES and TS generated the animals and performed the experiments. ES, JW, KS, SS, DB, TS, CE, KE, and BD wrote, edited, and prepared the manuscript. All authors contributed to the article and approved the submitted version.

## Conflict of Interest

The authors declare that the research was conducted in the absence of any commercial or financial relationships that could be construed as a potential conflict of interest.
